# Supplementary material for: The association of chest radiographic findings and severity scoring with clinical outcomes in patients with COVID-19 presenting to the emergency department of a tertiary care hospital in Pakistan
Source: PLoS One. 2021 Jan 5;16(1):e0244886. doi: 10.1371/journal.pone.0244886 (PMC7785233; doi:10.1371/journal.pone.0244886)
Supplement: Attachment — Submitted filename: Response Letter - PLOS.docx [file pone.0244886.s001.docx]

Dear Editor,

Thank you to you and the reviewers for making the time to review our manuscript and providing us with valuable comments. All authors have discussed the feedback received. We have addressed them as required, and believe the quality of article has thus been significantly elevated. Please find below a point-by-point response to all Reviewer comments received.

* * * * * * * * * * *

**1. Is the manuscript technically sound, and do the data support the conclusions? The manuscript must describe a technically sound piece of scientific research with data that supports the conclusions. Experiments must have been conducted rigorously, with appropriate controls, replication, and sample sizes. The conclusions must be drawn appropriately based on the data presented.**

**Reviewer #1: Yes**

**Reviewer #2: Yes**

*Authors’ Response*: Thank you.

**2. Has the statistical analysis been performed appropriately and rigorously?**

**Reviewer #1: Yes**

**Reviewer #2: Yes**

*Authors’ Response*: Thank you.

**3. Have the authors made all data underlying the findings in their manuscript fully available? The PLOS Data policy requires authors to make all data underlying the findings described in their manuscript fully available without restriction, with rare exception (please refer to the Data Availability Statement in the manuscript PDF file). The data should be provided as part of the manuscript or its supporting information, or deposited to a public repository. For example, in addition to summary statistics, the data points behind means, medians and variance measures should be available. If there are restrictions on publicly sharing data—e.g. participant privacy or use of data from a third party—those must be specified.**

**Reviewer #1: Yes**

**Reviewer #2: Yes**

*Authors’ Response*: Thank you.

**4. Is the manuscript presented in an intelligible fashion and written in standard English? PLOS ONE does not copyedit accepted manuscripts, so the language in submitted articles must be clear, correct, and unambiguous. Any typographical or grammatical errors should be corrected at revision, so please note any specific errors here.**

**Reviewer #1: No**

**Reviewer #2: Yes**

*Authors’ Response*: Thank you for your comment. We have proofread our manuscript thoroughly and ensured that all the minor typographical and grammatical errors are corrected (highlighted in the text).

**5. Review Comments to the Author Please use the space provided to explain your answers to the questions above. You may also include additional comments for the author, including concerns about dual publication, research ethics, or publication ethics. (Please upload your review as an attachment if it exceeds 20,000 characters)**

**Reviewer #1: Thank you very much for your submission. This is a nice study, which could set a nice ground for future research on prognosis and outcome of COVID-19. As a general comment, language and grammar needs revision. It does not deter from the possibility to understand the text, but there are multiple typos. I would also review the format. The tables could be reviewed and perhaps combined; the format could be made homogeneous. The resolution and quality of images should be reviewed .**

*Authors’ Response*: Thank you for your appreciation and your feedback. We have proofread the manuscript thoroughly and corrected grammatical errors (highlighted in manuscript). We have also reviewed the format of Table 2 and shortened it by removing 2 rows (listing frequencies and percentages of the individual initial and highest CXR-SS) which we feel were redundant. Moreover, we have ensured the resolution of the images is 1200 dpi and have used the PACE tool to make sure they are appropriate. We hope that it is adequate now.

**Reviewer #2: This is an high quality article and I appreciate a lot the paper. Only some minor suggestions: Discuss the possible role of ultrasound already in use in other infectious diseases ex tuberculosis and HIV (see and cite Bobbio F, Focused ultrasound to diagnose HIV-associated tuberculosis (FASH) in the extremely resource-limited setting of South Sudan: a cross-sectional study. BMJ Open. 2019 Apr 2;9(4):e027179. DOI: 10.1136/bmjopen-2018-027179. PMID: 30944140; PMCID: PMC6500283.) Figure 2 is not easy to read for the quality of image. Please add image with high quality**

*Authors’ Response*: Thank you for your appreciation and your feedback. We have added the suggested Reference in our Introduction, and discussed the role of ultrasound as well: “The use of ultrasound (US) has been explored previously in the diagnostics of other infectious diseases (2). Some studies have considered the emerging role of portable US imaging of the lungs in COVID-19 detection, describing ultrasound findings such as B-lines, consolidation and thickened pleural lines (3). However, US has its limitations, as it is highly operator-dependent and difficult to perform in patients with severe disease in an intensive care unit (ICU) set-up. Moreover, the specificity of ultrasound in patients with COVID-19 presenting with early disease has also proven to be significantly lower as compared to chest x-ray and computerized tomography scans (4)”. Additionally, we have ensured the resolution of the images is 1200 dpi and have used the PACE tool to make sure they are appropriate. We hope that it is adequate now.
